# Supplementary figures and images for: Th17 Immunity in the Colon Is Controlled by Two Novel Subsets of Colon-Specific Mononuclear Phagocytes
Source: Front Immunol. 2021 Apr 28;12:661290. doi: 10.3389/fimmu.2021.661290 (PMC8113646; doi:10.3389/fimmu.2021.661290)

Supplementary Figure 1

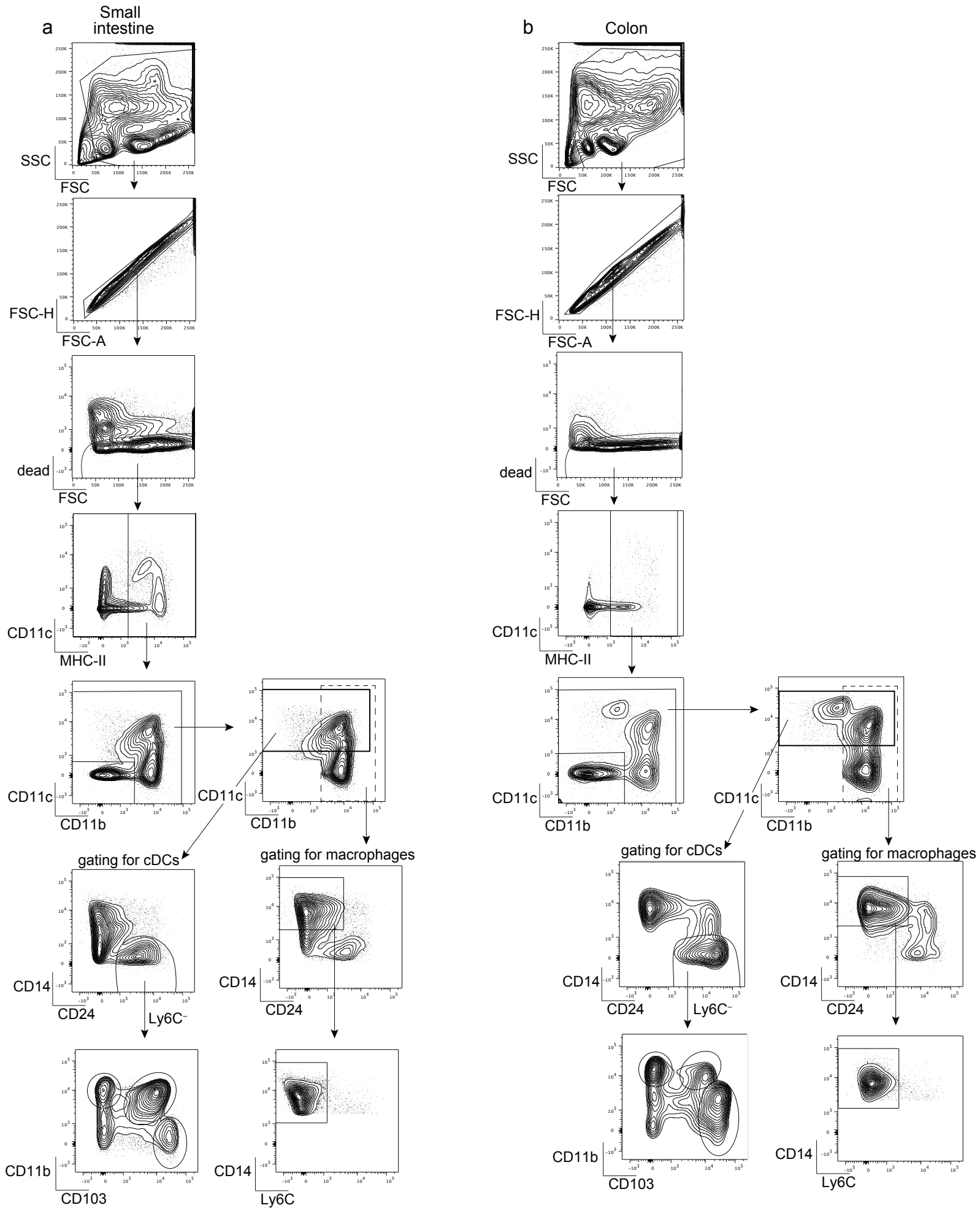

Supplement: Supplementary Figure 1 — Related to Figure 1 . (A, B) Gating strategy to identify CD103–CD11b+, CD103+CD11b–, and CD103+CD11b+ cDC subsets and conventional macrophages in lamina propria of small intestine (A) and colon (B). [file Image_1.pdf]

Supplementary Figure 2

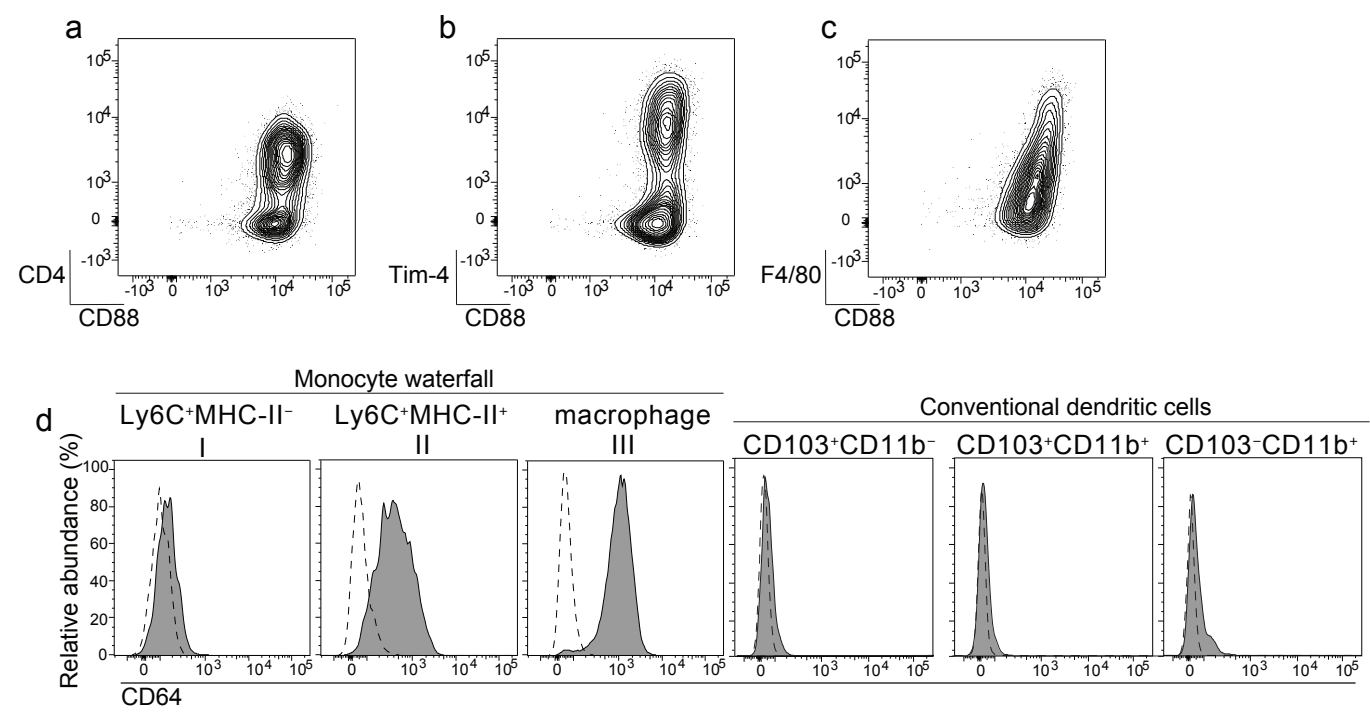

Supplement: Supplementary Figure 2 — Related to Figure 1 . Representative plot of small intestine lamina propria macrophages analyzed for CD88 expression together with CD4 (A), Tim-4 (B), or F4/80 (C). (D) Representative histograms of CD64 expression on the indicated populations of the monocyte-waterfall (gated as in Figure 1I ), macrophage, or cDC population. Dashed line: isotype control. [file Image_2.pdf]

Supplementary Figure 3

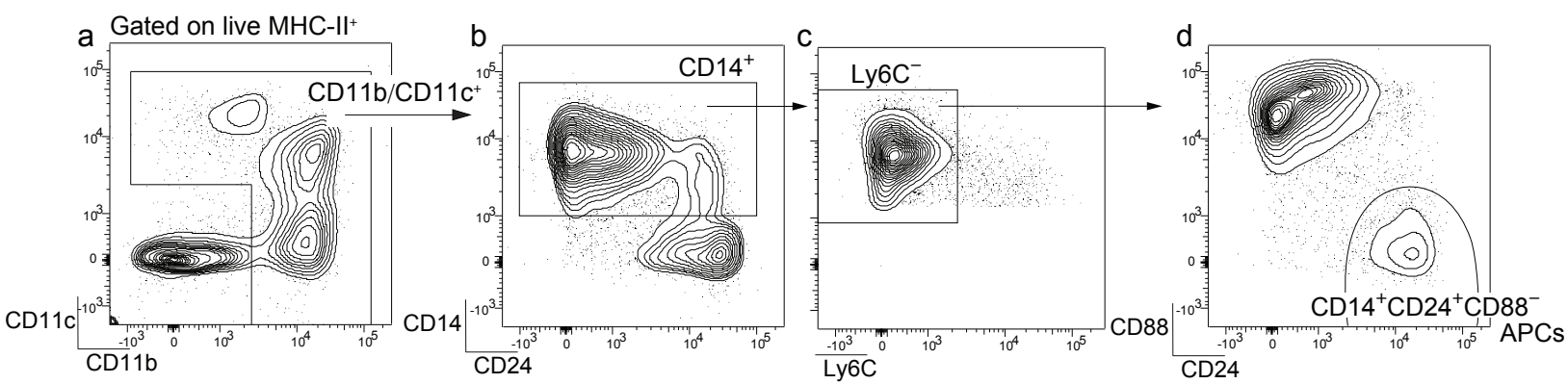

Gated population shown in panel (d)  
CD14<sup>+</sup>CD24<sup>+</sup>CD88<sup>-</sup> APCs

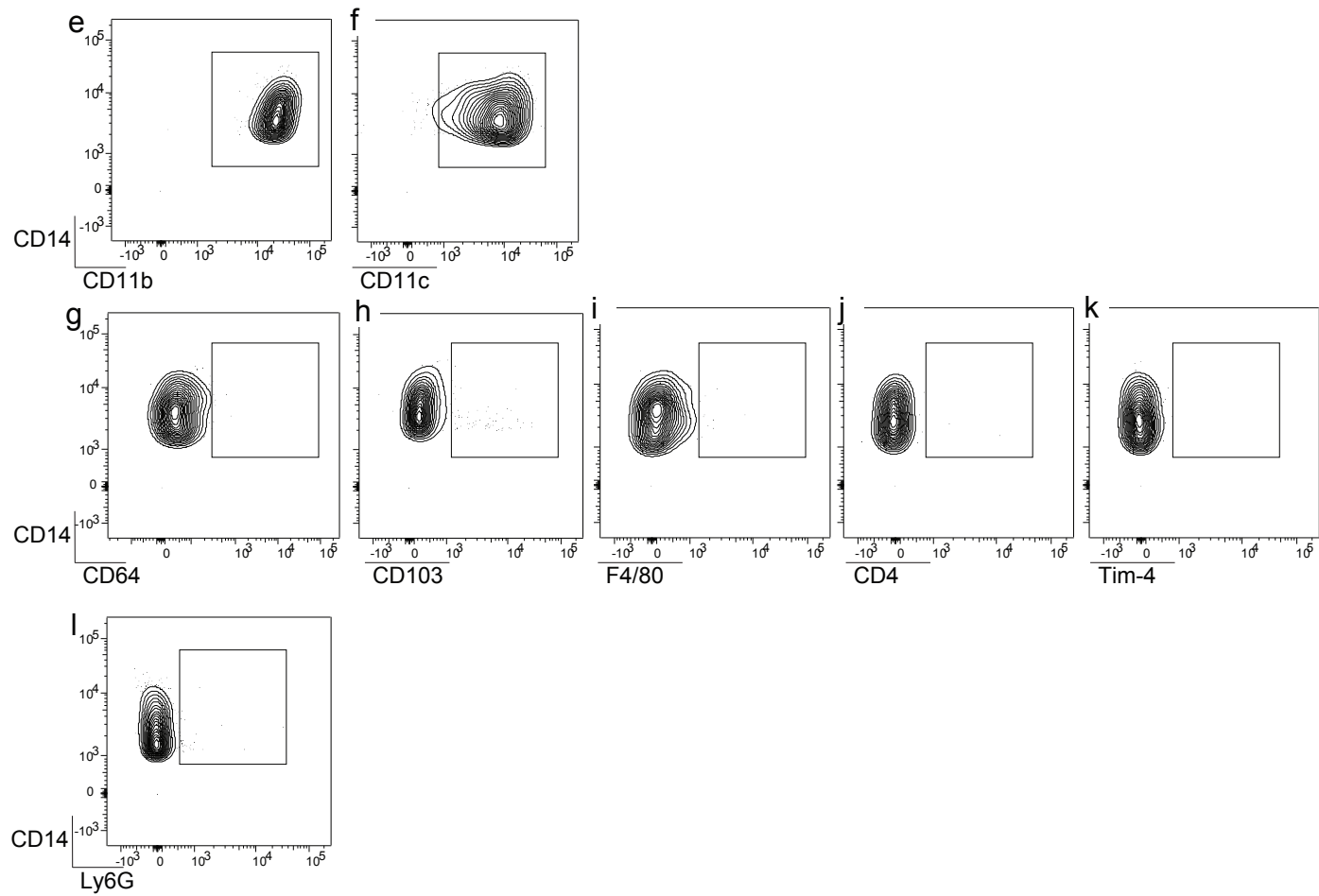

Supplement: Supplementary Figure 3 — Related to Figure 1 . (A–D) Gating strategy for C-LP CD14+CD24+CD88– APCs. Live MHC-II+ APCs as in Figure S1 were gated on populations expressing either CD11c or CD11b (A), followed by gating on CD14+ (B), and Ly6Cnegative populations (C). Gated populations in (C) were subsequently analyzed for CD88 and CD24 expression to distinguish CD14+CD24+CD88– APCs (D). Representative plot of C-LP CD14+CD24+CD88– APCs (gated in (D)) analyzed for expression of CD11b (E), CD11c (F), CD64 (G), CD103 (H), F4/80 (I), CD4 (J), Tim-4 (K), and Ly6G (L). [file Image_3.pdf]

Supplementary Figure 4

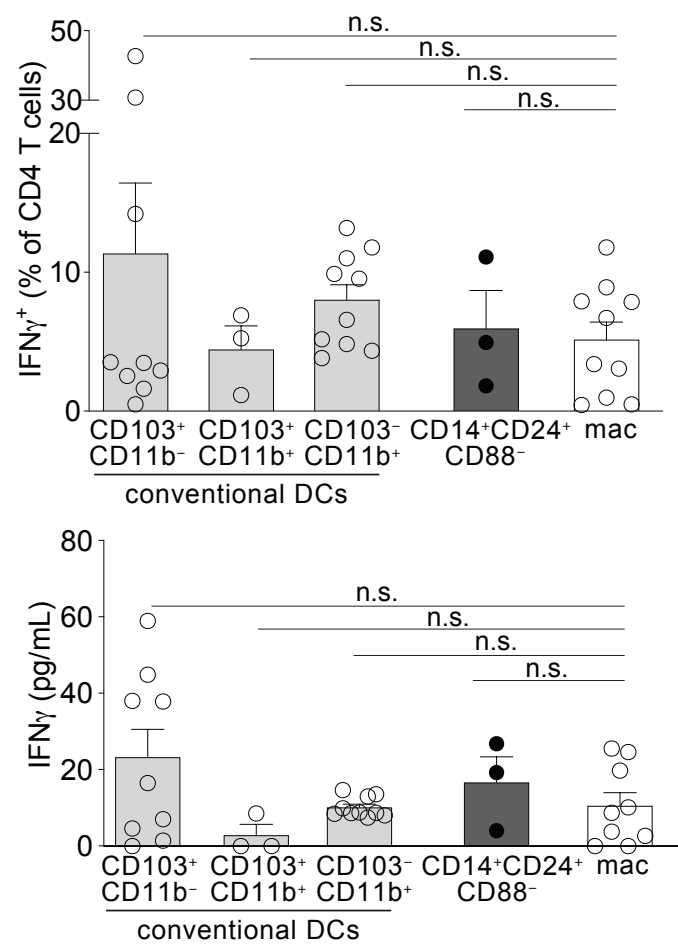

Supplement: Supplementary Figure 4 — Related to Figure 1 . Percentage of IFNγ+ OT-II T cells (A) and IFNγ protein (B) in supernatant of co-cultures described in Figure 1V . Each dot represents one experiment or replicates within an experiment. Error bars represent mean ± SEM. n.s. not significant (one-way ANOVA). [file Image_4.pdf]

## Supplementary Figure 5

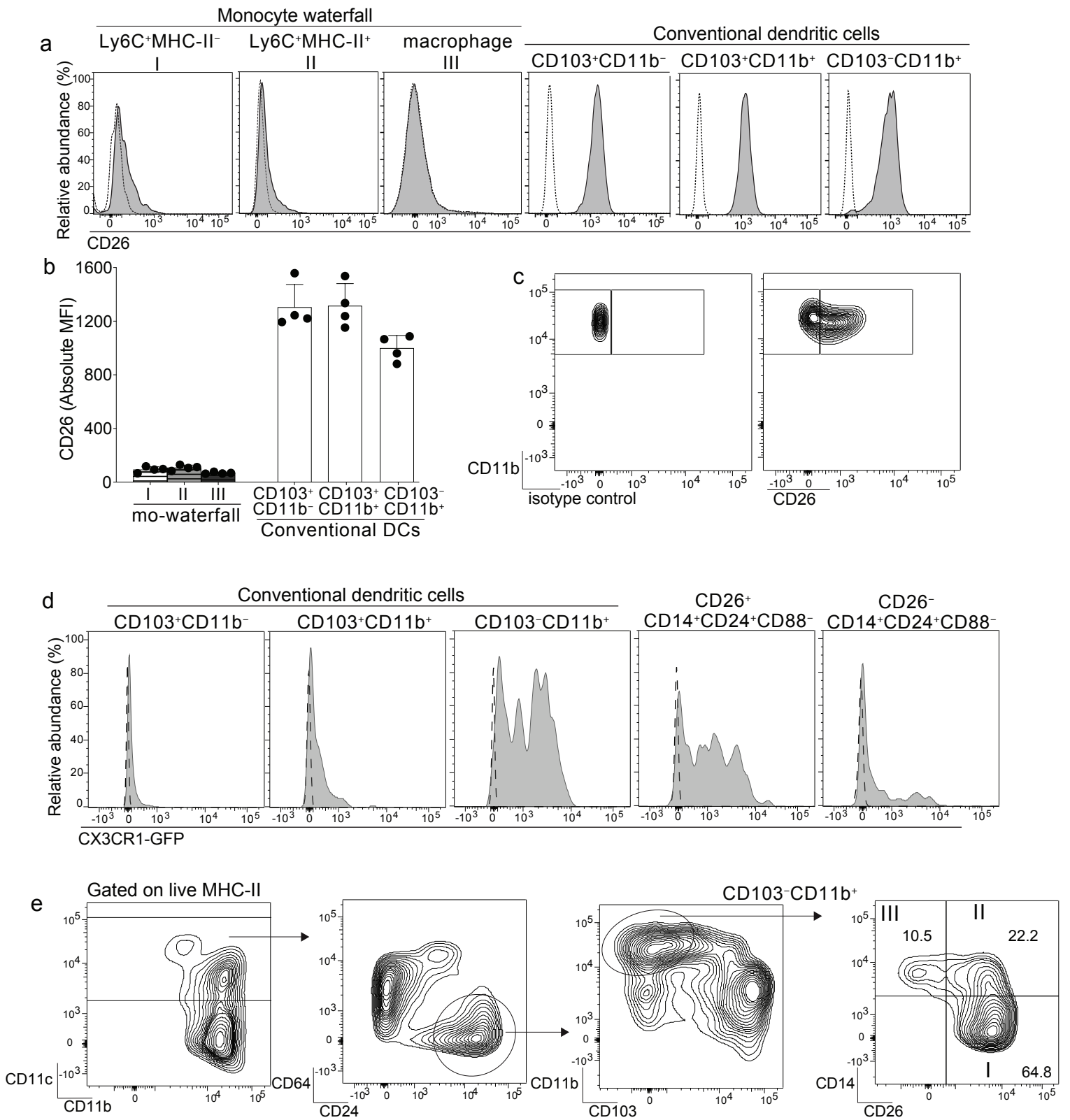

Supplement: Supplementary Figure 5 — Related to Figure 2 . (A, B) CD26 expression on the indicated C-LP mononuclear phagocyte population. Representative histogram (A) and absolute MFI (B) of CD26 expression on the indicated population. Dashed line: isotype control. Each dot in (B) represents one mouse. (C) Representative plot of isotype control or CD26 expression on C-LP CD14+CD24+CD88– APCs gated as in Figure 1U . (D) Representative histogram of CX3CR1-GFP expression on the indicated C-LP population. Dashed line: wild-type control. (E) Representative plot using conventional approaches to gating DCs (CD11c+CD24+CD64–) and the CD103–CD11b+ population within this refined gate. The CD103–CD11b+ population is heterogenous and can be further resolved into three subsets based on CD14 and CD26 expression: (I) CD103–CD11b+ DCs; (II) colon-specific CD26+CD14+CD24+CD88– APCs [as in (C)]; (III) colon-specific CD26–CD14+CD24+CD88– APCs [as in (C)]. [file Image_5.pdf]

Supplementary Figure 6

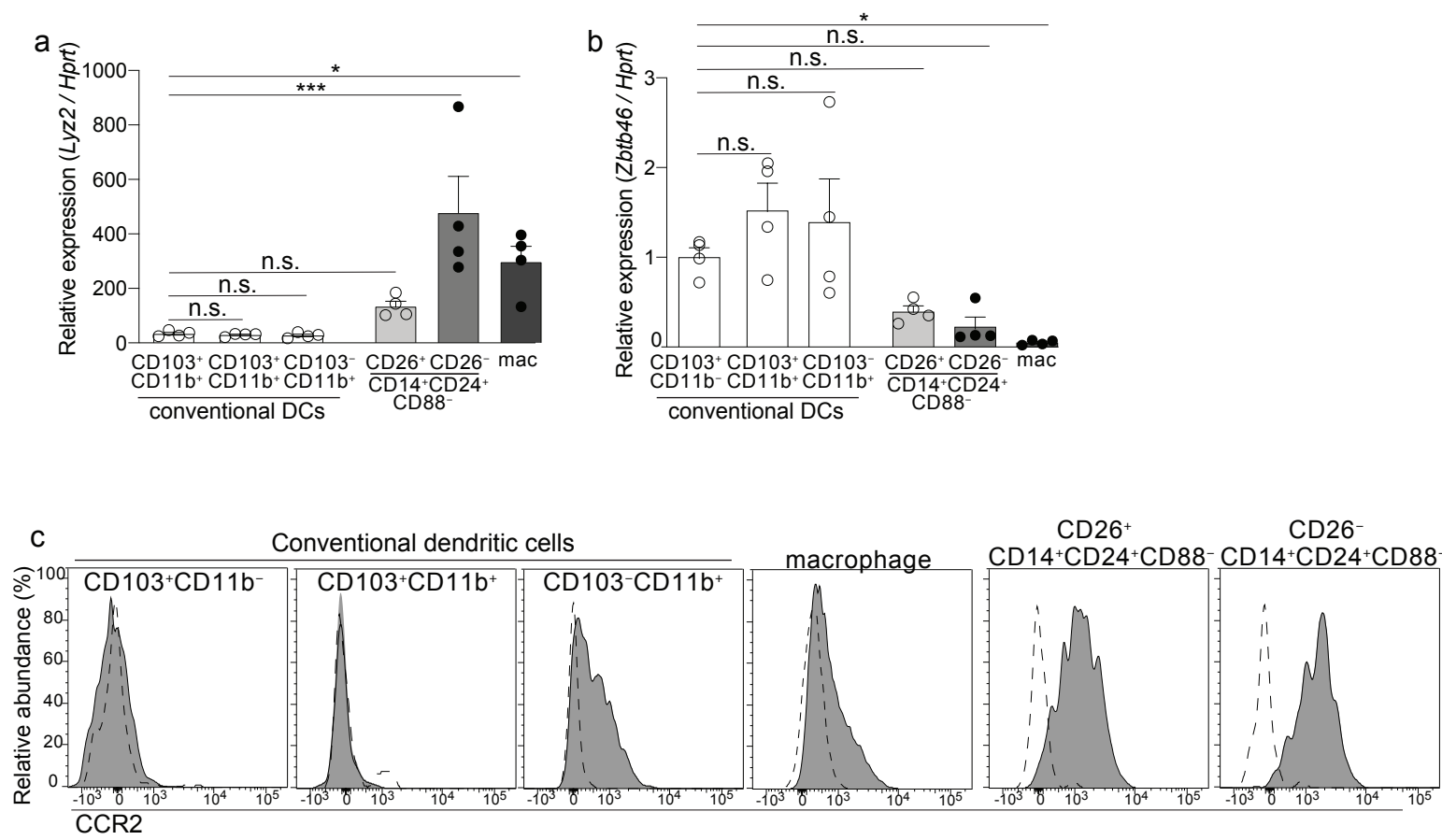

Supplement: Supplementary Figure 6 — Related to Figure 2 . (A, B) Gene expression of Lyz2 (A) or Zbtb46 (B) expression in the indicated C-LP population. Results are relative to expression of Hprt. Data was combined from 3-4 independent experiments each including pooled cells from at least 5 mice for each data point. (C) Representative histogram of CCR2 expression on the indicated C-LP mononuclear phagocyte population. Error bars represent mean ± SEM. *p < 0.05, ***p < 0.001, n.s. not significant (one-way ANOVA with Dunnett’s post hoc test contrasted to CD103+CD11b– cDCs). [file Image_6.pdf]

Supplementary Figure 7

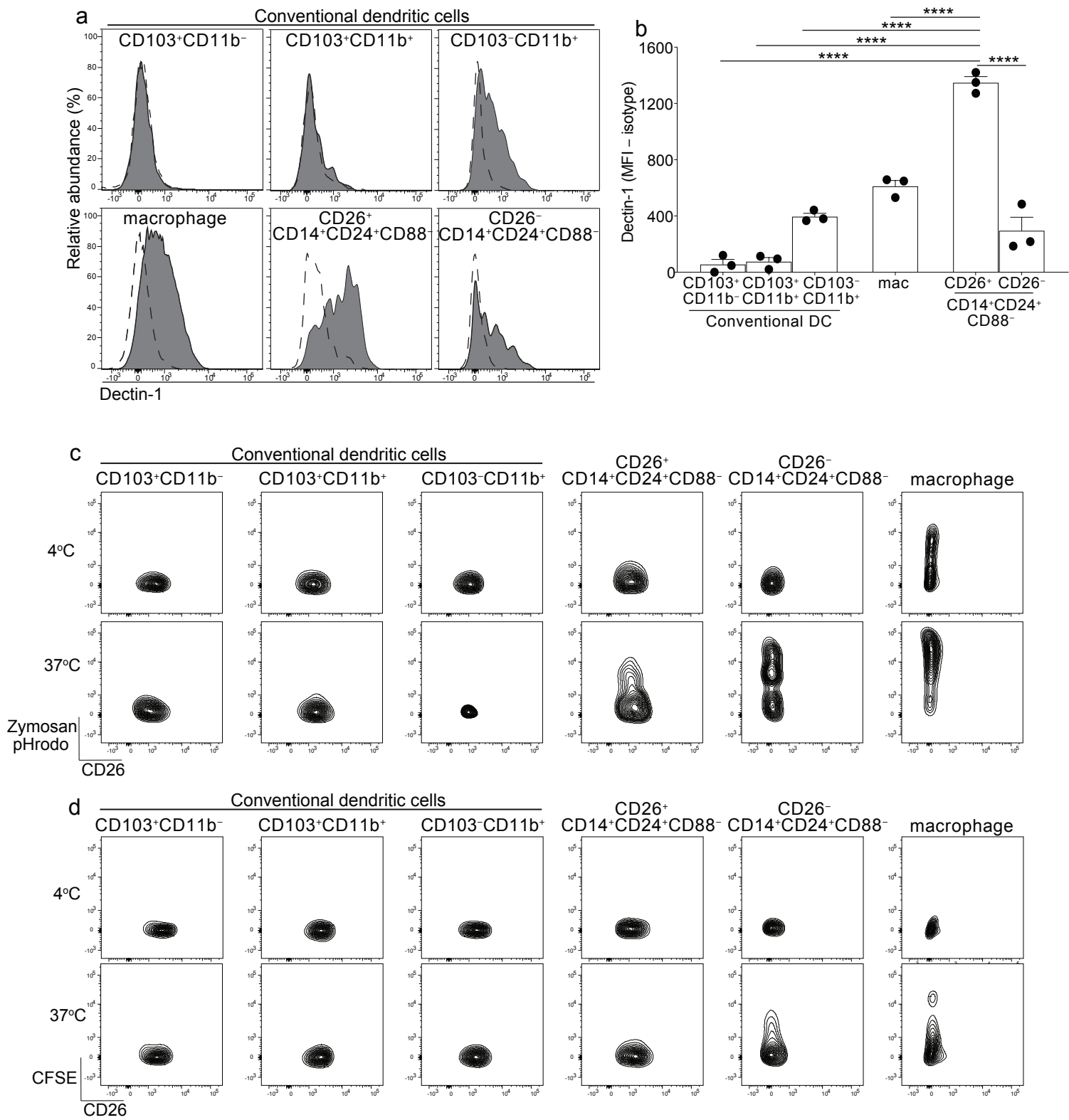

Supplement: Supplementary Figure 7 — Related to Figure 2 . (A, B) Surface Dectin-1 expression on the indicated C-LP mononuclear phagocyte subset. Representative histogram (A) and MFI for all mice tested (B). Dashed line: isotype control. Each dot in (B) represents one mouse. Error bars represent mean SEM. (C, D) Representative plot of zymosan pHrodo bioparticle™ fluorescence or CFSE in the indicated C-LP mononuclear phagocyte subset incubated with zymosan pHrodo bioparticles™ (C) or CFSE labeled apoptotic thymocytes (D) for 1h at 4°C or 37°C. Error bars represent mean ± SEM. ****p < 0.0001 (one-way ANOVA with Tukey’s post hoc test). [file Image_7.pdf]

Supplementary Figure 8

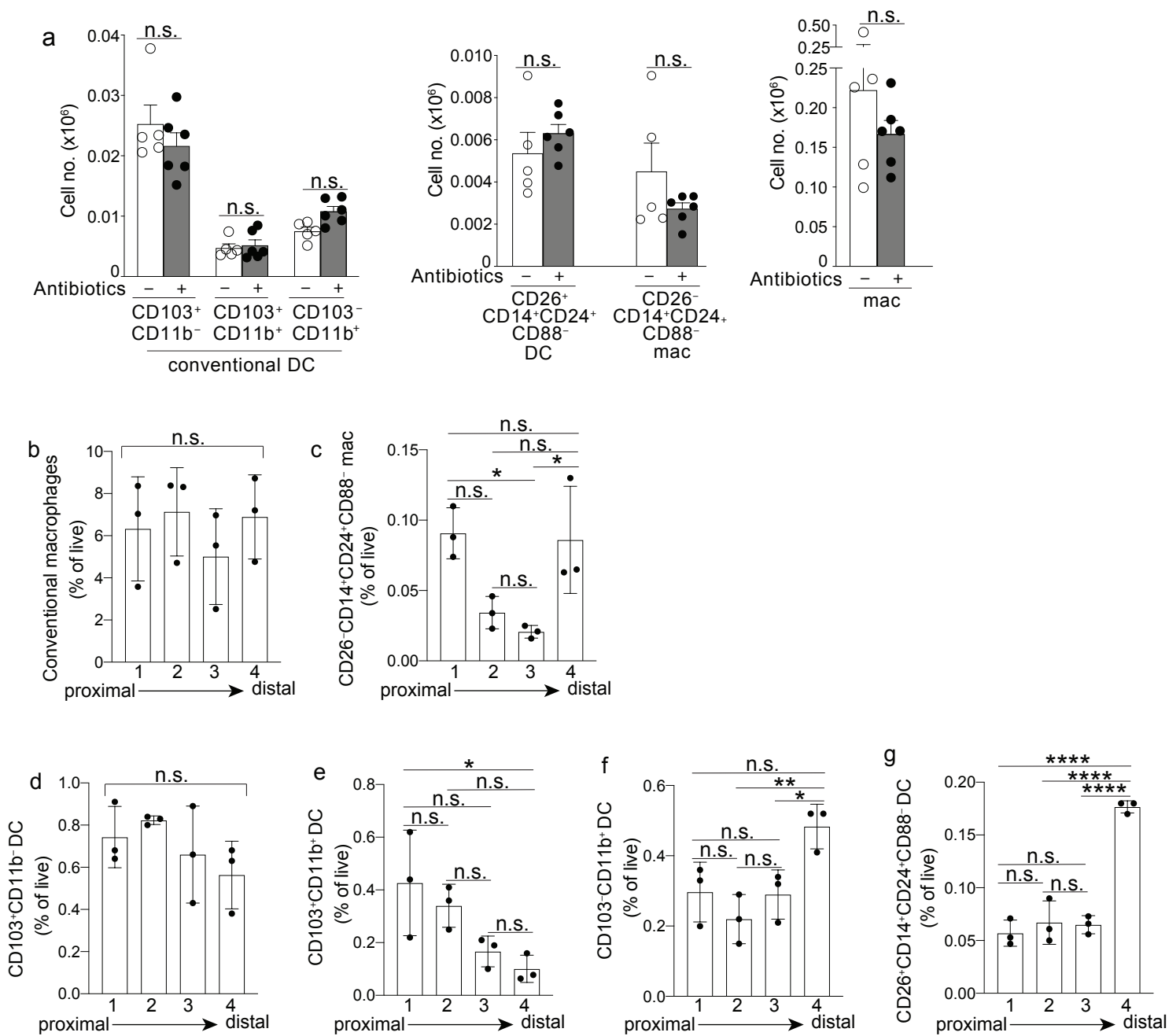

Supplement: Supplementary Figure 8 — Related to Figure 2 . (A) Cell numbers of the indicated mononuclear phagocyte population in eight week old mice administered broad spectrum antibiotics for four weeks. (B–G) The percentage of conventional macrophages (B), CD26–CD14+CD24+CD88– macrophages (C), CD103+CD11b– DCs (D), CD103+CD11b+ DCs (E), CD103–CD11b+ DCs (F), or CD26+CD14+CD24+CD88– DCs (G) among live cells isolated from the indicated quarter of the colon. Data is combined from three independent experiments each containing pooled cells from 4 mice, for each quarter segment. Error bars represent mean ± SD. *p < 0.05, **p < 0.01, ****p < 0.0001, n.s., not significant (one-way ANOVA with Tukey’s post hoc test). [file Image_8.pdf]

Supplementary Figure 9

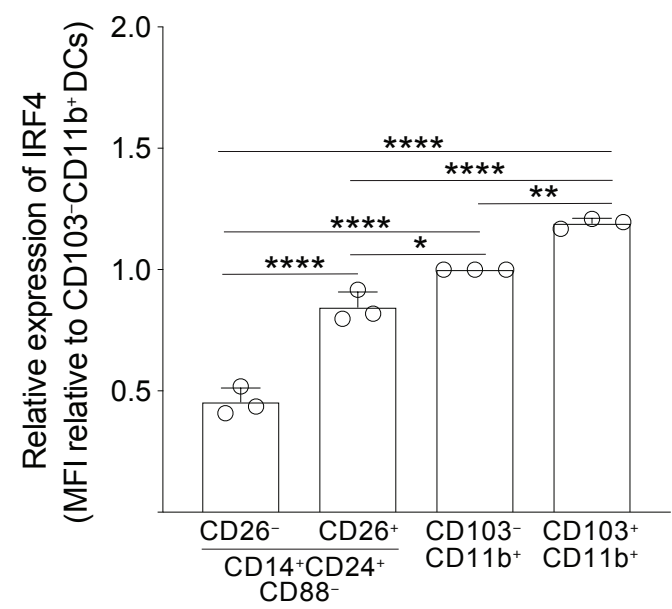

Supplement: Supplementary Figure 9 — Related to Figure 4 . Relative expression (MFI) of IRF4 protein in the indicated C-LP mononuclear phagocyte subset. Each dot represents one experiment containing pooled cells from at least 2 mice. Data is combined from three independent experiments. Error bars represent mean ± SEM. *p < 0.05, **p < 0.01, ***p < 0.001, ****p < 0.0001 (one-way ANOVA with Tukey’s post hoc test). [file Image_9.pdf]

Supplementary Figure 10

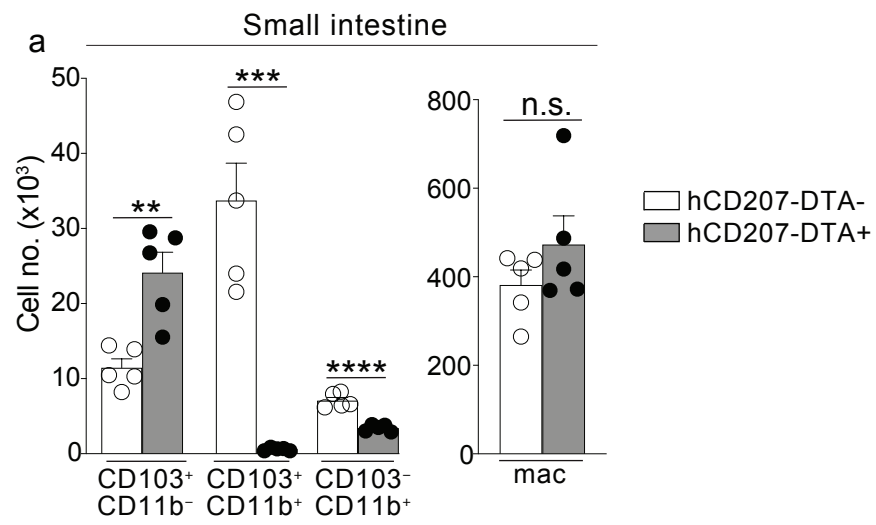

Supplement: Supplementary Figure 10 — Related to Figure 5 . Absolute cell number of the indicated small intestine lamina propria mononuclear phagocyte subset from co-housed, littermate hCD207-DTA– or hCD207-DTA+ mice. Each dot represents one mouse. Error bars represent mean ± SEM. *p < 0.05, **p < 0.01, ***p < 0.001, ****p < 0.0001, n.s. not significant (unpaired Student’s t test). [file Image_10.pdf]

Supplementary Figure 11: Graphical abstract

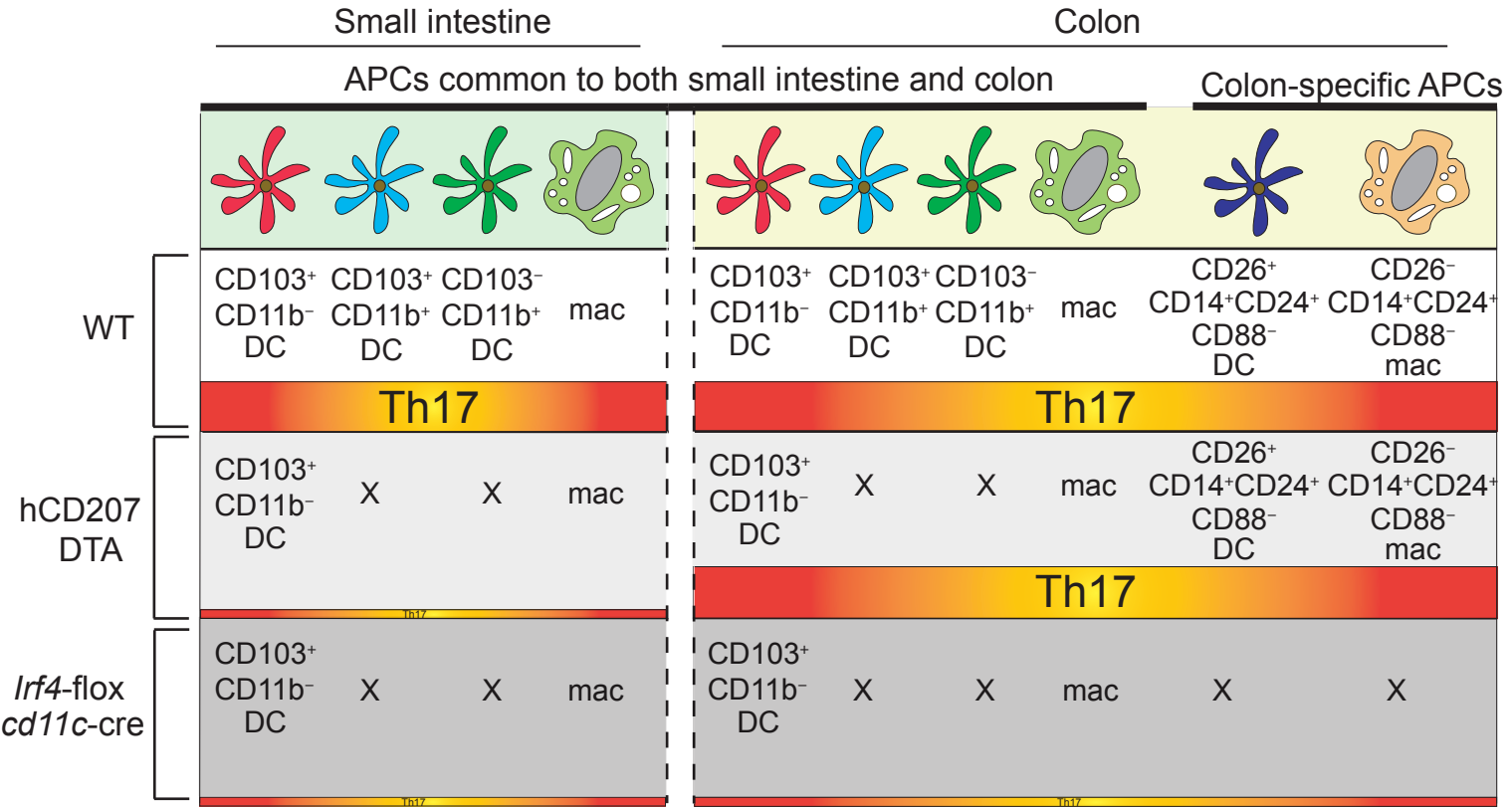

Supplement: Supplementary Figure 11 — Graphical abstract. DC and macrophage subsets of small intestine and colon, and Th17 cells in each intestinal organ in mice of the indicated genotype. Unlike other APC subsets, CD14+CD24+CD88– APCs are specific to the colon. Colon-specific CD14+CD24+CD88– APCs persist in hCD207-DTA mice, yet are ablated from Irf4-cko mice. This is unlike CD103+CD11b+ and CD103–CD11b+ DCs, which are depleted from both mouse strains. In the small intestine, CD103+CD11b+ and CD103–CD11b+ DCs are required for Th17 cells. This requirement is specific to small intestine, since colon-resident Th17 cells persist even in the absence of CD103+CD11b+ and CD103–CD11b+ DCs. Colon-resident Th17 cells instead require CD14+CD24+CD88– APCs, which are colon-specific. [file Image_11.pdf]
