# Supplementary material for: Th17 Immunity in the Colon Is Controlled by Two Novel Subsets of Colon-Specific Mononuclear Phagocytes
Source: Front Immunol. 2021 Apr 28;12:661290. doi: 10.3389/fimmu.2021.661290 (PMC8113646; doi:10.3389/fimmu.2021.661290)
Supplement: Supplementary file 12 [file Table_1.pdf]

Table S1

APC subset	Compare to	Genes Tested (Adjusted p value (significance))											
		Il6	*Il12b*	*Il23a*	*Il10*	*Tgfbr1*	*Ccl7*	*Mertk*	*Lyz2*	*Mmp13*	*Tnf*	*Zbtb46*	*Ccr7*
mac	$CD26^-$ $CD14^+CD24^+$ $CD88^-$ mac	0.0013 (**)	>0.9999 (ns)	0.9530 (ns)	<0.0001 (****)	0.0003 (***)	0.001 (***)	<0.0001 (****)	0.3182 (ns)	<0.0001 (****)	0.8683 (ns)	0.9948 (ns)	>0.9999 (ns)
mac	$CD26^+$ $CD14^+CD24^+$ $CD88^-$ DC	0.6413 (ns)	0.8118 (ns)	0.6194 (ns)	<0.0001 (****)	0.0002 (***)	0.0003 (***)	<0.0001 (****)	0.4220 (ns)	<0.0001 (****)	0.3492 (ns)	0.7783 (ns)	0.9913 (ns)
mac	$CD103^+$ $CD11b^-$ DC	>0.9999 (ns)	0.0477 (*)	>0.9999 (ns)	<0.0001 (****)	0.0011 (**)	0.0003 (***)	<0.0001 (****)	0.0585 (ns)	<0.0001 (****)	0.0294 (*)	0.0229 (*)	0.0192 (*)
mac	$CD103^+$ $CD11b^+$ DC	0.9317 (ns)	0.0166 (*)	0.0057 (**)	<0.0001 (****)	0.0006 (***)	0.0005 (***)	<0.0001 (****)	<0.0506 (ns)	<0.0001 (****)	0.2103 (ns)	0.0020 (**)	0.9010 (ns)
mac	$CD103^-$ $CD11b^+$ DC	0.8349 (ns)	0.0003 (***)	0.0042 (**)	<0.0001 (****)	0.0002 (***)	0.0002 (***)	<0.0001 (****)	<0.0525 (ns)	<0.0001 (****)	0.1792 (ns	0.0224 (*)	0.4536 (ns)
$CD26^-$ $CD14^+CD24^+$ $CD88^-$ mac	$CD26^+$ $CD14^+CD24^+$ $CD88^-$ DC	0.0329 (*)	0.8453 (ns)	0.9882 (ns)	0.9998 (ns)	0.9994 (ns)	0.9565 (ns)	0.7732 (ns)	0.0084 (**)	0.9999 (ns)	0.9133 (ns)	0.9809 (ns)	0.9978 (ns)
$CD26^-$ $CD14^+CD24^+$ $CD88^-$ mac	$CD103^+$ $CD11b^-$ DC	0.0014 (**)	0.0716 (ns)	0.9638 (ns)	0.3069 (ns)	0.9427 (ns)	0.9532 (ns)	0.9211 (ns)	0.0007 (***)	0.9999 (ns)	0.1799 (ns)	0.1863 (ns)	0.0259 (*)
$CD26^-$ $CD14^+CD24^+$ $CD88^-$ mac	$CD103^+$ $CD11b^+$ DC	0.0185 (*)	0.1360 (ns)	0.0468 (*)	0.4982 (ns)	0.9928 (ns)	0.9970 (ns)	0.9427 (ns)	0.0006 (***)	>0.9999 (ns)	0.7531 (ns)	0.0369 (*)	0.9452 (ns)
$CD26^-$ $CD14^+CD24^+$ $CD88^-$ mac	$CD103^-$ $CD11b^+$ DC	0.0147 (*)	0.0005 (***)	0.0435 (*)	0.8230 (ns)	>0.9999 (ns)	0.9189 (ns)	0.8287 (ns)	0.0006 (***)	0.9998 (ns)	0.6944 (ns)	0.0224 (*)	0.5385 (ns)
$CD26^+$ $CD14^+CD24^+$ $CD88^-$ DC	$CD103^+$ $CD11b^-$ DC	0.6364 (ns)	0.3638 (ns)	0.6530 (ns)	0.2002 (ns)	0.8224 (ns)	>0.9999 (ns)	0.9991 (ns)	0.8418 (ns)	>0.9999 (ns)	0.6264 (ns)	0.5762 (ns)	0.0615 (ns)
$CD26^+$ $CD14^+CD24^+$ $CD88^-$ DC	$CD103^+$ $CD11b^+$ DC	0.9936 (ns)	0.1360 (ns)	0.0966 (ns)	0.3506 (ns)	0.9461 (ns)	0.9988 (ns)	0.9977 (ns)	0.8061 (ns)	>0.9999 (ns)	0.9989 (ns)	0.1909 (ns)	0.9972 (ns)
$CD26^+$ $CD14^+CD24^+$ $CD88^-$ DC	$CD103^-$ $CD11b^+$ DC	0.9984 (ns)	0.0023 (**)	0.0920 (ns)	0.6748 (ns)	>0.9999 (ns)	>0.9999 (ns)	>0.9999 (ns)	0.8153 (ns)	>0.9999 (ns)	0.9964 (ns)	0.1279 (ns)	0.7886 (ns)
$CD103^+$ $CD11b^-$ DC	$CD103^+$ $CD11b^+$ DC	0.8517 (ns)	0.9629 (ns)	0.0063 (**)	0.9990 (ns)	0.9992 (ns)	0.9985 (ns)	>0.9999 (ns)	>0.9999 (ns)	>0.9999 (ns)	0.8222 (ns)	0.9672 (ns)	0.1433 (ns)
$CD103^+$ $CD11b^-$ DC	$CD103^-$ $CD11b^+$ DC	0.6364 (ns)	0.0794 (ns)	0.0048 (**)	0.9325 (ns)	0.8699 (ns)	>0.9999 (ns)	0.9999 (ns)	>0.9999 (ns)	>0.9999 (ns)	0.8694 (ns)	0.9052 (ns)	0.4977 (ns)
$CD103^+$ $CD11b^+$ DC	$CD103^-$ $CD11b^+$ DC	>0.9999 (ns)	0.3606 (ns)	>0.9999 (ns)	0.9919 (ns)	0.9685 (ns)	0.9944 (ns)	0.9995 (ns)	>0.9999 (ns)	>0.9999 (ns)	>0.9999 (ns)	0.9999 (ns)	0.9575 (ns)
